# Dust and Cobalt Levels in the Austrian Tungsten Industry: Workplace and Human Biomonitoring Data

**DOI:** 10.3390/ijerph13090931

**Published:** 2016-09-21

**Authors:** Hans-Peter Hutter, Peter Wallner, Hanns Moshammer, Gary Marsh

**Affiliations:** 1 Institute of Environmental Health, Center for Public Health, Medical University Vienna, 1090 Vienna, Austria; hans-peter.hutter@meduniwien.ac.at (H.-P.H.); peter.wallner@meduniwien.ac.at (P.W.); 2 Medicine and Environmental Protection, 1080 Vienna, Austria; 3 Department of Biostatistics, Graduate School of Public Health, University of Pittsburgh, Pittsburgh, 15261 PA, USA; gmarsh@pitt.edu

**Keywords:** biomonitoring, cobalt, hard metal industry, monitoring, tungsten

## Abstract

In general, routine industrial hygiene (IH) data are collected not to serve for scientific research but to check for compliance with occupational limit values. In the preparation of an occupational retrospective cohort study it is vital to test the validity of the exposure assessment based on incomplete (temporal coverage, departments) IH data. Existing IH data from a large hard metal plant was collected. Individual workers’ exposure per year and department was estimated based on linear regression of log-transformed exposure data for dust, tungsten, and cobalt. Estimated data were back-transformed, and for cobalt the validity of the estimates was confirmed by comparison with individual cobalt concentrations in urine. Air monitoring data were available from 1985 to 2012 and urine tests from the years 2008 to 2014. A declining trend and significant differences among departments was evident for all three air pollutants. The estimated time trend fitted the time trend in urine values well. At 1 mg/m^3^, cobalt in the air leads to an excretion of approximately 200 µg/L cobalt in urine. Cobalt levels in urine were significantly higher in smokers with an interaction effect between smoking and air concentrations. Exposure estimates of individual workers are generally feasible in the examined plant, although some departments are not documented sufficiently enough. Additional information (expert knowledge) is needed to fill these gaps.

## 1. Introduction

Cobalt (Co) is an essential element with ubiquitous dietary exposure. Further significant possible exposures are through occupation and medical devices. Focus of adverse health effects is the carcinogenic potential of Co (lung cancer). The metal and its compounds have been classified as carcinogenic since the 1970s, with a general International Agency for Research on Cancer (IARC) [1] assessment of cobalt in 1991 (group 2B). In its Monograph 86, IARC [2] rated cobalt metal with tungsten carbide as being probably carcinogenic to humans (group 2A). French [3,4,5] and Swedish [6] occupational cohort studies suggest that cobalt increased the risk of lung cancer—even more so in combination with tungsten carbide—in the hard metal industry. Additionally, there is evidence that hard metal particles exert genotoxic and carcinogenic activity [7,8].

Hard metal dust is also a known respiratory toxicant with chronic exposure leading to scarring of the lung tissue with consecutive restrictive and also obstructive lung disease [9,10,11]. Other nonrespiratory adverse health effects (cardiomyopathy, vision or hearing impairment) were observed at peak blood Co urine concentrations >700 µg/L (8–40 weeks), and reversible hypothyroidism and polycythemia were reported in humans at ~300 µg/L and higher (≥2 weeks) [12].

Cobalt is also classified as carcinogenic according to Austrian law [13]. As a result, a technical reference concentration (TRK) is specified instead of a health-based limit value (MAK). The TRK value, as a shift average, is set at 0.5 mg/m^3^ in respirable dust in the carbide industry and in the production of magnets, cobalt powder, and catalysts. In all other industries/areas a more stringent limit of 0.1 mg/m^3^ applies. A particular danger of skin absorption and the risk of respiratory and dermal sensitization are also pointed out in that legal document. The latter Austrian limit value is equal to the occupational limit value stipulated by the Occupational Safety and Health Administration (OSHA) (0.1 mg/m^3^) for General Industry (1910.1000 TABLE Z-1) or Construction Industry (29 CFR 1926.55 Appendix A), but the recommended exposure limit (REL) provided by the National Institute for Occupational Safety and Health (NIOSH) is lower (0.05 mg/m^3^), as is the threshold limit value (TLV) suggested by the American Conference of Governmental Industrial Hygienists (ACGIH) in 2001 (0.02 mg/m^3^). The same low value (0.02 mg/m^3^) has been stipulated as permissible exposure limit by the Californian OSHA (Table AC-1).

According to the regulation on health surveillance in the workplace [14], exposed workers must be tested annually for exposure to cobalt. For the urine test, a limit of 10 µg/L cobalt is defined. In case of its exceedance, another monitoring is mandatory after 6 months, but no further consequences are stipulated. Also, mandatory lung-function tests are performed annually. Unfortunately, in this study we had no access to these data but in another study [15,16] we have analysed repeated mandatory spirometric data obtained by a single Austrian occupational health center. In that case we did not focus specifically on hard metal dust, and only few workers had this specific exposure. Nevertheless, it was evident from the data that they performed rather poorly.

In principle, the employer must ensure that the air at the workplace is free of hazardous substances or, if that is not achievable, that the concentrations are kept as low as possible. This should also ensure that the internal exposure of workers, such as indicated by the excretion of harmful substances or their metabolites in urine, remains low. The rationale for assessing the internal exposure is basically twofold:

(1) In case of other important exposure pathways (e.g., skin or oral absorption) biomonitoring is the method of choice to control for these.
(2) In case of difficulties in monitoring the breathing air (e.g., in case of high spatial and temporal variability of the concentration) urine data can serve as a proxy for respiratory exposure.

In both cases, it makes no sense to stipulate a limit in urine that is exceeded even when the limit value in the air is met and no dermal (or oral) exposure occurs. If, on the other hand, stricter limit values for the urine are feasible this would indicate that also lower limit values in the air would be feasible and limit values for carcinogens should solely be based on technical feasibility.

Under the simplifying assumption of a “steady state”, which is valid in case of a constant exposure over a sufficiently long period, the uptake is equal to the excretion. When only inhalation is considered, the uptake depends on the concentration in the air, the breathing volume per minute, the duration of working shift (8 h, usually), and the percent retention of the dust in the airways. The dust particles contained in the air we breathe are partially deposited in the upper airways and partially exhaled, and the respective percentage depends on the particle size distribution. The particles deposited in the respiratory tract are transported partly via the mucociliary clearance to the pharynx and swallowed. Afterwards, cobalt is partly absorbed in the intestine but is also excreted in the stool. Assuming a concentration of 0.5 mg·cobalt/m^3^ and a ventilation of 10 L/min typical for light work over a working day of 8 h, a dose of 2400 µg cobalt is inhaled, of which about half (i.e., 1200 µg) is absorbed. About half of this is excreted via the kidneys and the remainder via the bile and, to a lesser extent, through the sweat. Assuming a daily urine volume of 2 L leads to a urine concentration of 300 µg/L. The same air–urine association is reported by the German Permanent Senate Commission for the Investigation of Health Hazards of Chemical Compounds in the Work Area [17,18,19]. Both the simple steady state model outlined above and the German formula indicate that the Austrian TRK-value is too high by more than an order of magnitude if compliance with the limit value (in urine) is sought. Therefore, we wanted to study the association between cobalt in dust and in urine in an Austrian data set.

An experimental approach concerning carcinogenic substances with targeted exposure of subjects is ethically problematic. Therefore, to estimate the association between cobalt in the air of the workplace and in post-shift urine samples, it is necessary to study routine data. This data is not primarily collected for answering research questions, but to verify compliance with legal limits. The data from a large Austrian plant, which has been collected as part of the preparatory work for an international retrospective cohort study [20,21] lends itself to this investigation. The following questions had to be answered in preparation for the cohort study: (1) Was the routine industrial hygiene (IH) data sufficient to estimate at least the ranking of cumulative exposures among the cohort members? (2) Could the validity of the IH data be supported by the biomonitoring data? (3) What was the relation between cobalt in the air and in urine? (4) Could we estimate temporal trends in exposure so that gaps in IH measurements, especially in earlier years, can be closed?

## 2. Methods

Initially, the work histories were collected from all workers who were hired at the Austrian plant since 1 January 1950. The industrial hygiene data of measurements of dust, tungsten, and cobalt were collected, and, in addition, biomonitoring data of cobalt in urine from exposed workers was obtained from the occupational health department. The evaluation of cobalt measurements in filter samples of the workplace air was hampered by differences in methodology and purpose of measurements. Of the 147 measurements on cobalt, 45 were personal and 102 area-related. Two of the measurements were carried out during the night shift, with only maintenance work and no full-scale operations. Only 134 measurements were carried out over the entire shift, while the rest had a significantly shorter (e.g., 1 h) averaging period. The shorter measurements mainly took place in the first study years. For 10 measurements the dust fraction is specified as “less than 5 microns”. For the remaining measurements information is missing in this regard. Numerous different reference methods are mentioned. The limit of quantification declined over the years from 0.01 to 0.002 mg/m^3^. A total of 17 samples were below the respective quantification limit. All air measurements were performed by the ÖSBS (Austrian Dust Control Association), the institution officially mandated with IH measurements in Austria by the Austrian workers’ compensation board (AUVA). They claim (personal communication Dipl. Ing. Jaschouz Daniel, 3 May 2016) that the different methods (different averaging times, pumps, air volumes, and filter materials) did not affect the results and that all methods were equivalent and validated.

Values below the quantification limit were set to “half the quantification limit”. In most instances, dust, tungsten, and cobalt had been measured in parallel. In those cases, pairwise correlations were analyzed and it was further examined whether the cobalt and tungsten content in dust varied among departments or exhibited a linear time trend. To that end, linear regression models were built with cobalt or tungsten as the dependent variable and dust as the independent variable, plus interaction terms with year or with “department” (as dummy variables).

IH data exhibited a markedly skewed distribution and were therefore log-transformed before further analysis. Log-levels of dust, cobalt, and tungsten were explained by a linear time trend, by analytical method, and department. “Analytical method” in that case stands for a set of parameters including personal or area-related measurement, type of pump and filters used, analytical instruments to measure the load on the filters, duration of sampling, and night versus day shift. This regression model allowed predicting log levels of exposures at other times at each department as well. The point estimates (for month and department) were then converted back from logarithmic to linear levels for the next step.

Cobalt in urine data were obtained from the occupational health department. They had documented the data in a simple excel sheet. In that documentation they did not discern between data below and at the detection limit of 1 µg/L. Spot samples were collected at the end of a shift. All urine analyses were performed at the laboratory of the Arbeitsmedizinischer Dienst (AMD, Occupational Health Service) in Linz: An iCE 3000 Series ETAA Spectrometer (Thermo Fisher Scientific, Loughborough, UK) equipped with a GFS35Z autosampler was used. The measurements were performed in pyrolytically coated tubes with a cobalt hollow cathode lamp (Cathodeon, Cambridge, UK; selected wavelength: 240.7 nm). High purity argon (99.999%) was applied as the protective and purge gas. Urine samples (100 µL) were diluted with 200 µL modifier (0.12% HNO_3_, 0.40% (NH_4_)2HPO_4_, 0.20% Triton-x-100; Reagents from Merck, Darmstadt, Germany, and of analytical grade). Volumes of 20 μL of diluted sample were injected into the graphite furnace.

Predicted exposures of cobalt were entered as explanatory variable for urine values in a second exploratory linear regression model. This model also included current smoking status and the examination month to control for residual confounding by time trend. We assessed the interaction between (current) smoking and cobalt in dust as well. When first preliminary analyses showed that persons with missing information regarding current smoking status had urine values similar to nonsmokers, these two groups were merged in final analyses. The final analyses were in the form of random-effects generalised least squares (GLS) regression with the group variable either defined by job-class or by worker.

All data were analyzed with STATA 13.1 SE (StataCorp, College Station, TX, USA). The study was approved by the ethics committee of the Medical University of Innsbruck (AN2014-0380 345/4.9 from 30 January 2015).

## 3. Results

The following data was available: air measurements to dust (*n* = 130), tungsten (*n* = 141), and cobalt (*n* = 147) for the years 1985–2012 and urine tests of 253 people (a total of 1166 records) from the years 2008 to 2014. Persons were anonymised but job descriptions were provided. Table 1 presents the basic data.

Cobalt and tungsten were each highly and significantly correlated with dust (*r* = 0.95 and 0.54, respectively). Per mg/m^3^ of dust, cobalt concentration (air) increased by 0.11 mg/m^3^ (95% CI: 0.1–0.11). The corresponding values for tungsten were 0.21 (0.15–0.27). This ratio did not vary significantly among the departments although for some departments there were not enough data points for reasonable estimates. Assuming the same dust levels, tungsten levels declined over time by 0.06 mg/m^3^ (0.02–0.11) annually. This was not seen with cobalt. Assuming the same dust levels, tungsten values were lower in personal compared to area-level samples (0.02 mg/m^3^ per mg/m^3^ compared to 0.24; *p* for interaction = 0.015). Contrary to that, cobalt levels were higher in personal samples (0.19 compared to 0.1; *p* < 0.001).

**Figure 1:**
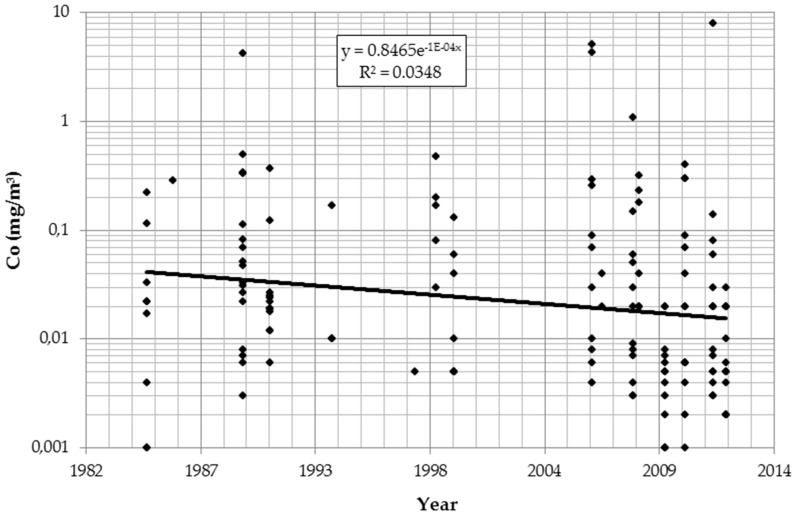
Time trend of cobalt (Co) in air (log scale).

The logarithmic values of all IH parameters (dust, cobalt, and tungsten) significantly declined over time and differed by department. Table 2 describes the findings for cobalt after controlling for details of analytical procedures. Results for dust and tungsten were similar (data not shown). Figure 1 presents the temporal trend in cobalt concentration irrespective of department and measurement strategies.

Based on the results of the regression analysis (Table 2) for time trend (monthly values) and department, cobalt exposures in air were estimated for each urine sample. Few urine samples (114 of 1166) were provided by workers working in departments with no information from IH-measurements. For these, exposures were assumed to be equal to departments with similar tasks. Some people worked in administration (16 samples) or the lab (37 samples). Their airborne exposure was set to 0, but dropping these data points did not alter the point estimates substantially (data not shown). Even fewer workers (22 samples) were working in maintenance at the time of urine collection. Their exposure was deemed too variable to be estimated accurately and their data were excluded from further calculations. The estimated exposures (excluding the estimates of 0) clearly displayed a biphasic pattern indicating the inclusion of departments with low (around 0.02 mg/m^3^) and with somewhat higher cobalt exposure (around 0.1 mg/m^3^).

**Figure 2:**
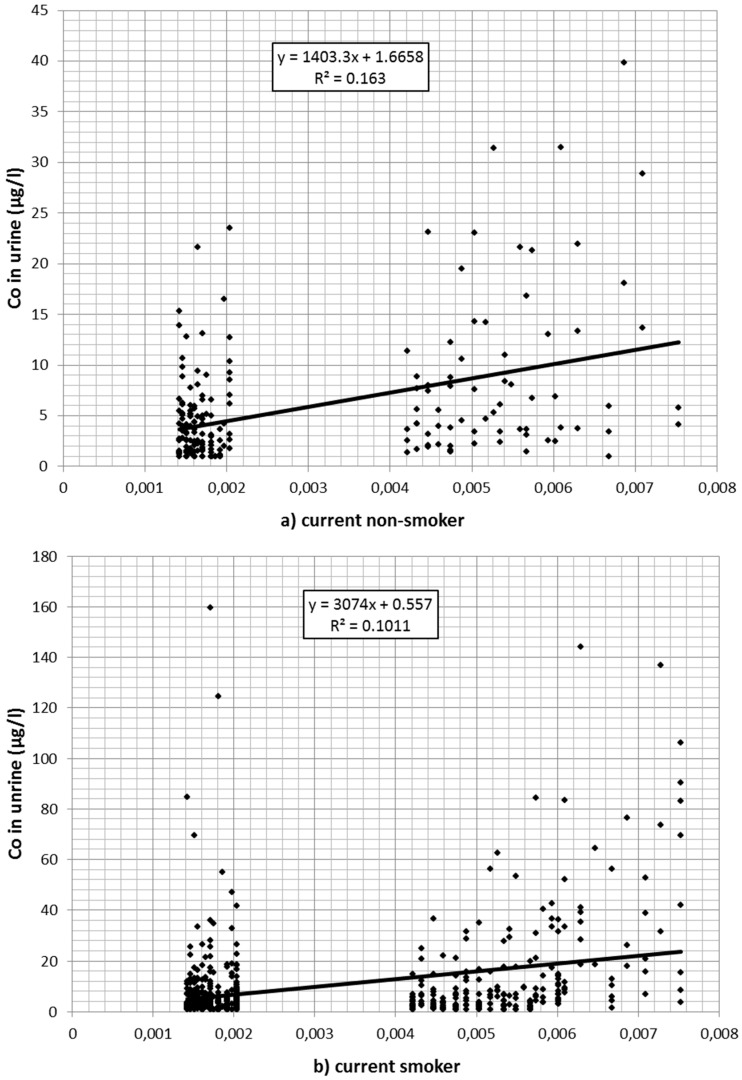

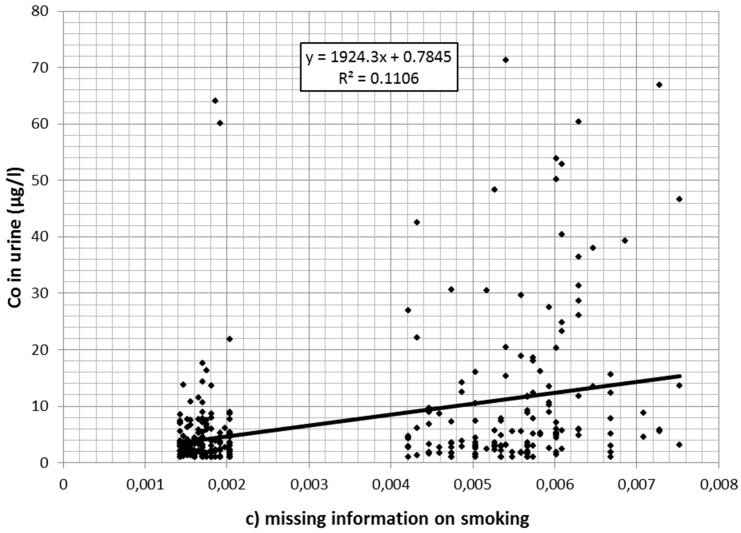
(**a**) Association between cobalt (Co) in the air (estimated from regression analysis) and cobalt in urine for (current) nonsmokers; (**b**) association between cobalt in the air (estimated from regression analysis) and cobalt in urine for smokers; (**c**) association between cobalt in the air (estimated from regression analysis) and cobalt in urine for workers with missing smoking information.

Also, cobalt concentration in urine declined over time. Of the 1166 values 139 were reported to be at or below 1 µg/L. A total of 229 values exceeded the Austrian limit value of 10 µg/L. Cobalt in the air (estimated from the regression model described in Table 1 after back-transformation from logarithmic values) was associated with cobalt values in urine (Table 3). Figure 2a–c depicts the association between (estimated) cobalt in the air and cobalt in urine for current smokers (a); nonsmokers (b); and for workers without information on current smoking (c) separately.

Workers with missing information on smoking did not differ significantly from nonsmokers in the preliminary model. Therefore, it was assumed that most of these were nonsmokers or at least light smokers only. In an additional regression model, they were merged with the nonsmokers. The significantly higher cobalt burden in smokers is plausible considering the additional oral exposure via the dust–hand cigarette–mouth path. In that case an interaction between smoking and cobalt in the air would be expected: The higher the occupational exposure, the stronger the additional effect of smoking. Indeed, in the interaction model only a small direct effect of smoking per se was found (1.89 µg/L per mg/m^3^, *p* = 0.316). The impact of cobalt in air (per mg/m^3^) was weak in nonsmokers (79.46 µg/L, *p* < 0.001), while in smokers the urine concentration (per mg/m^3^ in the air) increased by an additional 403.21 µg/L, *p* < 0.001).

In the final panel models (Table 3) there remained a significant negative time trend and the analyses indicated a relevant part of the variance to be between panels (25% for job class and 49% for individual workers).

## 4. Discussion

Overall, work histories were collected from 1968 workers. Repeated urine tests were available from 253 of those workers for the years 2008–2014. Air measurements of dust (*n* = 130), tungsten (*n* = 141), and cobalt (*n* = 147) exist for the years 1985–2012. For all substances a significant decrease over the years is detectable. The reduction of occupational exposures over the years has been reported from many industries, countries, and substances [22,23,24,25]. The annual reduction in airborne cobalt exposures in this Austrian plant is in the same order of magnitude. This is reassuring, considering the observation of increasing rates of sensitization against cobalt in persons of working age [26]. The authors of that paper suggest that increasing occupational exposures could play a role. This hypothesis is not supported by the Austrian data.

The air and urine values of cobalt correlate with each other: for 1 mg/m^3^ cobalt in the air, there was an excretion of approximately 200 µg/L cobalt in urine (panels by workers). This ratio differs by smoking status. The Austrian occupational limit value for cobalt (0.5 mg/m^3^) is too lenient compared to the limit in urine (10 µg/L). According to our data, a concentration of 0.5 mg/m^3^ in the workplace air would lead to a urine concentration of at least 100 µg/L. Even assuming the stricter limit value in other industries (0.1 mg/m^3^) we would still expect a urine concentration of more than 20 µg/L. Most of the urine concentrations were below the Austrian limit value indicating that concentrations in the air well below the current Austrian limit values are technically feasible. This is underlined by a recent Swedish study as well [27], which reported cobalt concentrations (arithmetic mean, range) of 0.0030, 0.000028–0.056 mg/m^3^.

The available data allow the initial assessment of the exposure of these worker cohorts. The correlations between the different IH parameters (dust, cobalt, and tungsten) on the one hand are reassuring. On the other hand, it prevents a separate analysis of the effects of cobalt alone compared to mixed exposures. Both air and urine measurements indicate a decreasing trend over time overall. However, the data from the Austrian plant alone are not sufficient to extrapolate the exposure to earlier time periods and to some other departments or job classes.

The study made use of routine data collected for legislative and administrative purposes. Because of data protection and privacy issues, many personal details of the participants were not available. The data analysis was further hampered by incomplete data (e.g., on smoking behavior) and by a huge variation in measurement methodologies over time. The regression analysis indicated that most of these differences did not significantly affect the results and dropping these parameters did not substantially change the point estimates for time trend and department effects. This finding is in line with the claim by the ÖSBS that all the methods used over the years were equivalent. Only personal versus area-level sampling affected the other point estimates, although in the case of cobalt the concentrations found by the two approaches did not differ significantly. Nevertheless, for future research and documentation purposes it is recommended to stick to a single measurement scheme and keep data in an easily accessible way.

## 5. Conclusions

This paper adds to the ongoing public health debate in Austria regarding the occupational limit values for cobalt. These Austrian limit values are not based on health considerations but purely on technical feasibility. Although in some instances these limit values have been exceeded, in general and on average the cobalt concentrations in the workplace air were far below the current limit values. In order to ensure compliance with existing limit values for cobalt in urine, stricter limit values for the air would also be necessary.

This paper also provided valuable information for the ongoing cohort study. In spite of remaining uncertainties and data gaps, the findings will help to improve exposure estimates for all cohort members.

## Figures and Tables

**Table 1:** Descriptive statistics.

**Cohort**	**Number**	**Percent**
Total Cohort	1969	100
White collar workers	331	16.9
Males	1550	78.7
Still working in 2014	656	33.3
Austrian Citizens	1683	85.5
**Cohort**	**Arithmetic Mean**	**Range**
Year of birth	1965	1908–1994
Year at first hire	1990	1946–2012
Age at first hire	25.3	14–57
Duration of work (years)	16.6	0–67
**IH-Data (Number)**	**Median**	**Range**
Dust (140)	0.3 mg/m^3^	0.04–75
Cobalt (147)	0.02 mg/m^3^	0.001–8
Tungsten (141)	0.1 mg/m^3^	0.004–37.3
**Urine Data**		
Number of persons	253	
Number of samples	1166	
**Urine Data**	**Median**	**Range**
Cobalt (µg/L)	3.7	1–159.7

IH: Industrial Hygiene.

**Table 2:** Results of linear regression: Time trend and differences per department in ln(cobalt) concentration levels after controlling for various analytical details. Note that for some departments there are only very few data points rendering the point estimates imprecise.

**Factor**		**Point Estimate**	***p*-Value**
Trend per year		−0.047	0.023
Personal sampling		0	(reference)
Area level		−0.508	0.217
**Department**	**Measurements (Number)**		
Extrude	(19)	0	(reference)
Press	(19)	0.297	0.595
Shape	(74)	1.124	0.029
Furnace	(1)	−0.715	0.685
Grind	(1)	4.428	0.018
Drill, mill, bore	(1)	0.735	0.681
Powder room	(27)	1.369	0.009
Graphite service	(5)	2.398	0.009
Constant		87.79	0.032

**Table 3:** Results of linear regression: association between cobalt in urine (µg/L) and in the air (mg/m^3^). Effect of (current) smoking.

**Factor**	**Point Estimate**	***p*-Value**
Cobalt in air (mg/m^3^)	315.25	<0.001
Nonsmoker	0	(reference)
Smoker	4.09	<0.001
Missing information	1.2	0.335
Constant	4.92	<0.001
Cobalt in air (mg/m^3^)	317.94	<0.001
Nonsmoker and missing	0	(reference)
Smoker	3.36	<0.001
Constant	5.65	<0.001
**Panels by Job Class**	**Point Estimate**	***p*-Value**
Cobalt in air (mg/m^3^)	167.33	0.037
Time (days)	−0.0017	0.003
Nonsmoker and missing	0	(reference)
Smoker	3.93	<0.001
Constant	40.76	<0.001
rho	0.25	-
**Panels by Worker**	**Point Estimate**	***p*-Value**
Cobalt in air (mg/m^3^)	206.52	<0.001
Time (days)	−0.002	<0.001
Nonsmoker and missing	0	(reference)
Smoker	2.54	0.73
Constant	43.17	<0.001
rho	0.49	-
